# An Epidemic of Dengue-1 in a Remote Village in Rural Laos

**DOI:** 10.1371/journal.pntd.0002360

**Published:** 2013-08-08

**Authors:** Audrey Dubot-Pérès, Phengta Vongphrachanh, Justin Denny, Rattanaphone Phetsouvanh, Singharath Linthavong, Bounthanom Sengkeopraseuth, Amphai Khasing, Vimattha Xaythideth, Catrin E. Moore, Manivanh Vongsouvath, Josée Castonguay-Vanier, Bountoy Sibounheuang, Thaksinaporn Taojaikong, Anisone Chanthongthip, Xavier de Lamballerie, Paul N. Newton

**Affiliations:** 1 UMR_D 190 Emergence des Pathologies Virales, Aix-Marseille Université, IRD French Institute of Research for Development, EHESP French School of Public Health, Marseille, France; 2 Lao-Oxford-Mahosot Hospital - Wellcome Trust Research Unit (LOMWRU), Microbiology Laboratory, Mahosot Hospital, Vientiane, Lao People's Democratic Republic; 3 Centre for Tropical Medicine, Nuffield Department of Clinical Medicine, University of Oxford, Churchill Hospital, Oxford, United Kingdom; 4 National Centre for Laboratory and Epidemiology, Ministry of Health, Vientiane, Lao People's Democratic Republic; 5 World Health Organization – Lao People's Democratic Republic, Vientiane, Lao People's Democratic Republic; 6 Xayabury Provincial Health Department, Xayabury, Lao People's Democratic Republic; University of California, Davis, United States of America

## Abstract

In the Lao PDR (Laos), urban dengue is an increasingly recognised public health problem. We describe a dengue-1 virus outbreak in a rural northwestern Lao forest village during the cool season of 2008. The isolated strain was genotypically “endemic” and not “sylvatic,” belonging to the genotype 1, Asia 3 clade. Phylogenetic analyses of 37 other dengue-1 sequences from diverse areas of Laos between 2007 and 2010 showed that the geographic distribution of some strains remained focal overtime while others were dispersed throughout the country. Evidence that dengue viruses have broad circulation in the region, crossing country borders, was also obtained. Whether the outbreak arose from dengue importation from an urban centre into a dengue-naïve community or crossed into the village from a forest cycle is unknown. More epidemiological and entomological investigations are required to understand dengue epidemiology and the importance of rural and forest dengue dynamics in Laos.

## Author Summary

Dengue disease is caused by a virus transmitted by mosquitoes. In Southeast Asia, where it is endemic, it represents a very important public health problem. Major outbreaks, including severe cases and death, occur every year. Two distinct transmission cycles have been described. Most common is the human-mosquito-human cycle observed throughout most tropical regions of the world, often associated with urban locations and always human habitations, often producing explosive outbreaks, whereas “sylvatic” dengue, genetically different, circulates in forest wild animals and has been reported to be able to infect humans. In the Lao PDR, a developing country where dengue is endemic, data on this disease are sparse. This study reports an unusual outbreak of dengue that occurred during the cold season in a village in a forested area. It also is the first extensive analysis of dengue virus nucleotide sequences, from 39 patients across the country, from Laos. Results suggest three patterns of dengue circulation in Laos: local transmission, transmission over the whole country, and transmission implicating bordering countries. The dengue virus isolated from patients in the forest village outbreak proved to be genetically similar to those found in urbanized areas throughout the country. More investigations are needed to understand the relationships between dengue in forested and urban areas.

## Introduction

Dengue is endemic in more than 100 countries in Asia, Africa and the Americas, but 70% of those currently at risk live in South-East Asia and the Western Pacific. WHO estimates that 50–100 million people are infected by dengue globally every year [1]. Dengue infections may be asymptomatic or symptomatic, classified as dengue fever (DF), dengue haemorrhagic fever (DHF), and dengue shock syndrome (DSS) [2] or more recently as dengue, dengue with warning signs and severe dengue [3]. Dengue fever is characterized by a sudden onset of high-grade fever with non-specific symptoms and most cases resolve without specific treatment. However, DHF, caused by increased vascular permeability, may progress to hypovolaemic shock and to potentially lethal DSS [2]. Dengue viruses (DENV) are single stranded RNA viruses from the family *Flaviviridae*, transmitted by *Aedes* spp. mosquitoes, which are predominantly urban. Sylvatic dengue has also been described in humans in SE Asian and West African forests [4], [5], [6], [7], [8], [9], [10], [11] but has only been associated with one outbreak [5]. Although there is evidence of interactions between urban and sylvatic dengue, their importance for dengue epidemiology and public health is not well understood [11].

In the Lao PDR (Laos), dengue is endemic with re-occurring epidemics during the monsoon season [12], [13], [14], [15]. Diagnosis of dengue in Laos is usually based on clinical symptoms [15], biological confirmation being only occasional. This is a major public health issue as many other pathogens have clinical manifestation similar to those of dengue. There is little information on the eco-epidemiology of dengue in the country or whether rural populations are affected.

Here, we report an epidemic of dengue in a rural village of the Xayabury Province, north-west Laos in 2008, with the first dengue molecular epidemiology data from Laos, including dengue strains from across Laos from 2007 to 2010, and discuss their public health significance.

## Methods

**Figure 1:**
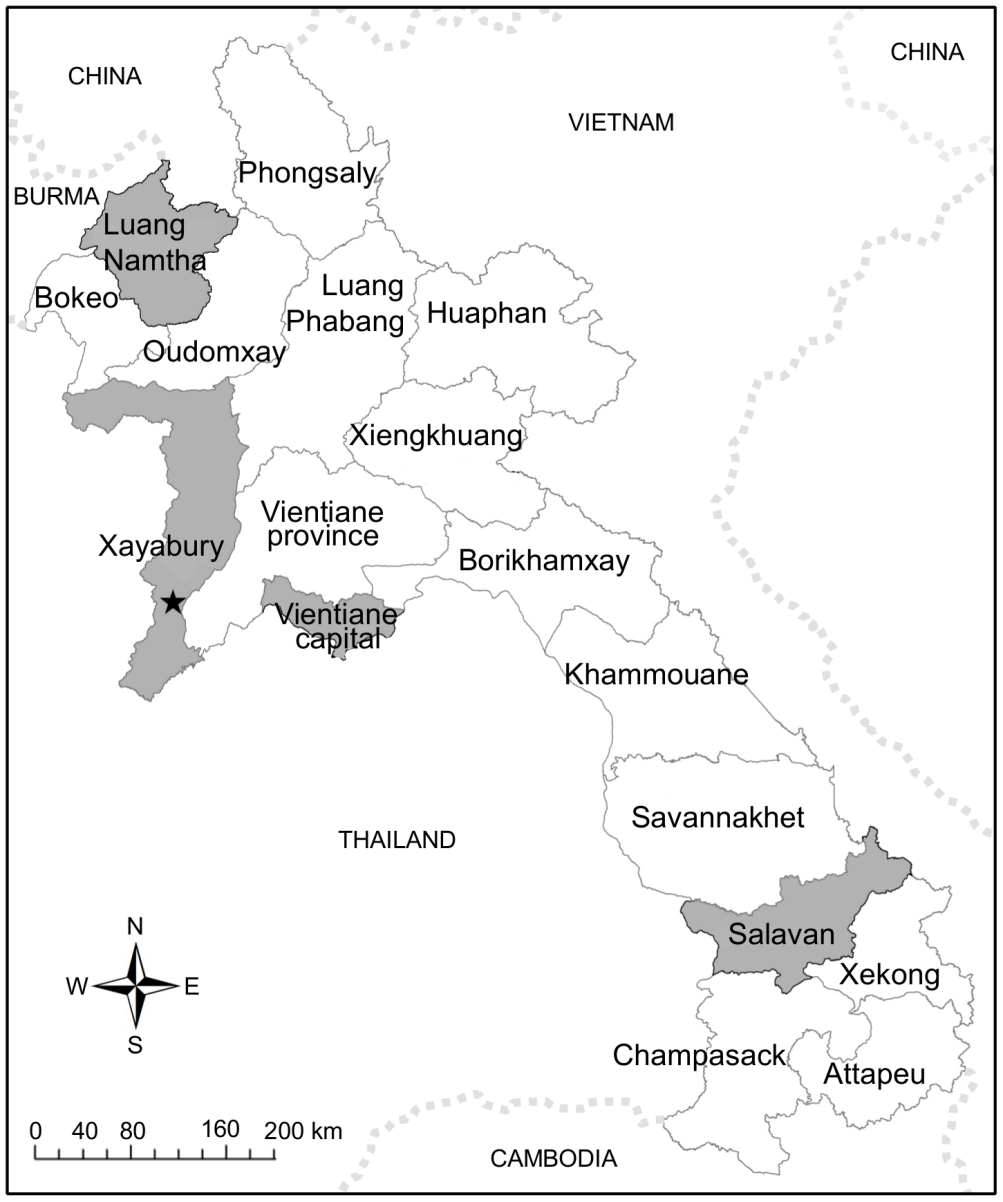
Map of Lao PDR. Locations of the hometowns of the patients whose dengue viruses were sequenced are in grey (Luang Namtha, Latsavang (indicated by a star), Vientiane and Salavan).

In November and December 2008, an outbreak of unexplained fever occurred in Latsavang Village (18.222°N, 101.322°E, altitude ∼312 m above msl), Paklai District, Xayabury Province, NW Laos (Figure 1). Latsavang village is on the bank of the Namyang River, 60 km, by forest track, from the nearest town of Pak Lai, 10 km to the E and 14 km to the W of the Lao/Thai border (Muang Chet Ton in Thailand). The 1,526 inhabitants, living in 298 households, are predominantly maize farmers.

### Patients' samples

#### From the outbreak

An investigation team from Xayabury Provincial Health Department, National Centre for Laboratory and Epidemiology (NCLE) and the World Health Organization (WHO) country office visited the village on 12^th^–20^th^ December 2008. Patients and their families were interviewed and examined. Sera from 11 patients, taken after oral informed consent as part of outbreak investigation, were sampled and sent at ambient temperature to Vientiane for analysis, arriving within 24 h of collection. Acute samples were taken from all eleven patients on December 15^th^, at the apparent end of the outbreak, and convalescent samples were obtained five days later on December 20^th^ from seven patients (Table 1).

**Table 1:** Clinical details of the 11 patients with blood samples, all survived.

	Sample collection date					
Sample number	Acute	Convalescent	Age (year)	Sex	Days of illness, signs and symptoms	Evidence for Dengue infection
XB998	14-Dec-2008	20-Dec-2008	5	M	1 day fever, headache, nausea, dizziness	Yes
XB999	15-Dec-2008	20-Dec-2008	8	M	1 day fever, headache, red eyes	Yes
XB1000	14-Dec-2008	20-Dec-2008	12	M	7 days of fever, headache. Nausea, dizziness	Yes
XB1001	14-Dec-2008	20-Dec-2008	10	M	3 days of fever, headache, myalgia, red face	Yes
XB1010	15-Dec-2008	20-Dec-2008	23	F	1 day of fever, headache, nausea, dizziness	Yes
XB1011	15-Dec-2008	N/A	15	M	1 day of fever, headache, vomiting	Yes
XB1012	14-Dec-2008	20-Dec-2008	12	F	3 days of fever, headache, vomiting, dizziness	Yes
XB1014	15-Dec-2008	N/A	32	M	1 day fever, headache, vomiting	Yes
XB1009	15-Dec-2008	20-Dec-2008	25	M	7 days fever and headache	No
XB1013	15-Dec-2008	N/A	41	M	1 day fever, headache, vomiting	No
XB1015	15-Dec-2008	N/A	52	F	1 day fever, headache, vomiting	No

N/A: data not available.

#### From other locations

Admission sera from 37 dengue patients collected in the framework of dengue research studies conducted at Lao-Oxford-Mahosot Hospital-Wellcome Trust Research Unit (LOMWRU) were used for phylogenetic analyses. Nineteen patients were admitted at Mahosot Hospital (Vientiane), 5 patients at Luang Namtha Provincial Hospital, and thirteen patients at Salavan Provincial Hospital (Figure 1), with unexplained fever between 2007 and 2010. Dengue 1 infection was confirmed for these patients by PCR. Written informed consent was obtained from these patients or their responsible guardians.

### Ethics statement

For the outbreak investigation, performed by the Government of the Lao PDR and WHO, oral informed consent was obtained from all patients (or their guardians/parents if they were aged <15 years). As this was an urgent public health outbreak investigation for diagnosis of the epidemic, ethical approval was not requested and oral consent was not formally documented. Retro-active ethical approval was not requested for the outbreak investigation.

All the patients seen at Mahosot Hospital, Luang Namtha Provincial Hospital and Salavan Provincial Hospital, with unexplained fever, gave informed written consent. For children, their guardians/parents gave informed written consent. These studies were granted ethical approval by the Lao National Ethics Committee for Health Research (124/NECHR, 134/NECHR) and the Oxford Tropical Research Ethics Committee (025-02, 006-07, 015-10)

### DNA and RNA extractions

DNA was extracted from 200 µl of acute serum using the QIAamp DNA minikit (Qiagen), following manufacturer's instruction, and eluted with 100 µl of elution buffer. Viral RNA was extracted using EZ1 Virus v2.0 Kit and EZ1 Biorobot (Qiagen) from 200 µl of acute serum following manufacturer's instruction and eluted with 90 µl of elution buffer. Internal phage controls were added to all sera before extraction to monitor the amplification processing and check for the presence of PCR inhibitors [16].

### Dengue diagnosis

Diagnosis of dengue relied on the detection of either *(i)* NS1 dengue antigen (Dengue Early ELISA (Cat no. E-DEN01P), PanBio Ltd., Sinnamon Park, Queensland, Australia), *(ii)* specific anti-dengue IgM antibodies (Japanese encephalitis-dengue IgM Combo ELISA (Cat no. E-JED01C), PanBio), *(iii)* high level specific anti-dengue IgG antibodies associated with acute secondary dengue infection (Dengue IgG capture ELISA (Cat no. E-DEN02G), PanBio), (iv) and/or dengue PCR. ELISA tests were performed following manufacturer's instruction. During secondary dengue infection, anti-dengue IgM antibodies may be produced at low or undetectable levels, whereas anti-dengue IgG reach levels above those found in primary or past infection [2]. The Dengue IgG capture ELISA kit (Panbio) permits specific detection of these high levels of anti-dengue IgG antibodies present in acute secondary infection, increasing sensitivity for detecting dengue, when IgM and/or NS1 are not detected.

Random Reverse Transcription (RT), using hexamer primer, was performed using 10 µl of RNA extracts and the TaqMan Reverse Transcription Reagents (Applied Biosystems) following the manufacturer's instruction in a final volume of 50 µl.

The ‘Dengue All’ TaqMan real-time PCR (that detects all 4 dengue serotypes) was performed on 10 µl of RT product, following Leparc-Goffart *et al.* [17] and using the Platinum Quantitative PCR SuperMix-UDG kit (Invitrogen). Samples positive with this PCR were further characterised by using serotype-specific real time PCR systems [17].

The Panflavivirus SYBR Green real-time PCR [18], that detects all viruses belonging to the genus *Flavivirus* (family *Flaviviridae*), was performed using 3 µl of RT product and the QuantiMix Easy SYG kit (Biotools, Madrid, Spain). Amplicons (272 bp in the NS5 gene) were sequenced (Macrogen Inc., Seoul, Korea) and the corresponding sequences were BLASTed on the NCBI website (blastn) for identification. All negative primary Panflavivirus PCR reactions underwent a hemi-nested PCR using 3 µl of the primary PCR product, the same reverse primer, a distinct degenerate forward primer [19] and the same amplification protocol as in the primary PCR. Amplicons (200 bp) were sequenced and BLASTed for identification.

In a Biosafety Level 3 laboratory, 200 µl of patients' sera were inoculated onto confluent Vero cells in a 12 well plate format. After 1 week at 37°C in a 5% CO_2_ incubator, cells were scraped off and centrifuged. The clarified supernatant (SN) was collected and 1 ml was passaged onto fresh Vero cells using 12.5 cm^2^ flasks. To check for dengue virus growth for all patient sera, viral RNA was extracted from 0.2 ml of SN at passages 0 (inoculation on plates) and 1 (12.5 cm^2^ flasks) using the QIAamp MinElute spin kit (Qiagen) following manufacturer's instructions with an elution volume of 90 µl, and tested using the dengue 1-specific real time PCR system and Panflavivirus real-time PCR, as described above [17], [18].

### Dengue 1 genome sequencing

Eighteen PCR amplification systems (Table S1), distributed along the complete dengue 1 (DENV-1) genome (from bases 17 to 10,737), were designed from the alignment of complete genome sequences of 36 DENV-1 strains isolated worldwide (GenBank accession numbers in supporting material Text S1). They were used to produce overlapping PCRs along the viral genome from random hexamer RT reactions. The PCRs were performed using 3 µl of RT with 1.25 unit of AmpliTaq Gold DNA polymerase (Applied Biosystems), 2 mM of MgCl_2_, 200 µM of dNTPs and 400 nM of each primer. The 3′ end fragment of the genome was amplified using the QuantiTect SYBR Green RT-PCR Kit (Qiagen) directly from 5 µl of RNA extract. The corresponding PCR products were sequenced by Macrogen Inc.

Nearly full-length DENV-1 genome sequences were produced directly from the sera of 7 patients: two patients from Latsavang, 2 patients from Northern Laos (Luang Namtha, October 2008 and September 2009), 2 patients from Southern Laos (Salavan, September and October 2008) and one patient from Vientiane (December 2008).

For sera from 32 additional DENV-1 patients from Luang Namtha, Salavan and Vientiane, between 2007 and 2010, 1,723 bases covering the dengue 1 envelope gene were amplified and sequenced using 3 pairs of primers (Table S1).

### Phylogenetic and recombination analyses

All 2,160 DENV-1 envelope sequences available in the Hemorrhagic Fever Viruses (HFV) database (http://hfv.lanl.gov) were downloaded and aligned, using ClustalX2.1 [20], with the 39 Lao DENV-1 envelope sequences obtained in this study. A Neighbour-Joining tree was constructed using Mega 5.05 software with Kimura-2 model [21]. Reliability of nodes was assessed by bootstrap resampling with 500 replicates. Another tree using the Maximun Likelihood method was constructed using the same alignment.

In addition to this ‘exhaustive’ reconstruction, another Neighbour-Joining tree was constructed from the alignment of the 39 Lao envelope sequences with the 1,318 complete or nearly complete DENV-1 genomic sequences (10,000 bases or more) available in HFVdb, to produce a tree with a robust phylogenetic backbone.

The 1,318 complete (or nearly complete) DENV-1 genomic sequences were aligned, using ClustalX2.1, to the 7 Lao DENV-1 genome sequences obtained in this study. Indication for the presence of molecular recombinations was investigated using the Recombination Detection Program (RDP) version 3 software [22]. RDP, GENCONV and MAXCHI methods were used for primary screening and the BOOTSCAN and SISCAN methods for automatic checking of the recombination signals [23], [24], [25], [26], [27]. For optimal recombination detection, we selected the RDP3 automask procedure that allows automatic masking of all sequences except one within groups of similar sequences. This means that within each group of similar sequences, only one sequence was examined during the exploratory search for recombination signals. When the program finds a recombination signal in an unmasked sequence it then examines all masked sequences from the same group (RDP user manual, http://darwin.uvigo.es/rdp/rdp.html).

Recombination events of more than 400 bases with a multiple comparison corrected p-value lower than e-10 were selected for downstream phylogenetic analyses. Neighbour-Joining trees using Mega 5.05 software with Kimura-2 model were produced from the alignment of the sequences remaining after the automask process. For each recombination event, two trees were produced and compared: one using sequences located between the putative recombination breakpoint positions and another excluding the putative recombinant region.

### Other aetiological investigations

#### Japanese encephalitis virus (JEV) serological detection

The Japanese encephalitis-dengue IgM Combo kit used for dengue allowed the simultaneous detection of IgM anti-JEV antibodies (Cat no. E-JED01C, PanBio) [28].

#### Scrub typhus (*Orientia tsutsugamushi*) and murine typhus (*Rickettsia typhi*) serology and PCR

Acute and convalescent sera were tested by immunofluorescence assays (IFA) for the presence of IgM and IgG antibodies to *O. tsutsugamushi* (indicating scrub typhus infection) and *R. typhi* (indicating murine typhus) as described in Phetsouvanh et al. [29]. A positive result was defined as either an IgM or IgG titer >400 for scrub typhus or murine typhus according to the criteria suggested for Thailand by Coleman *et al.* [30] or a fourfold rise in titre between acute and convalescent sera when available.

A SYBR Green real-time PCR technique [31], targeting *groEL* gene, was used to detect *O. tsutsugamushi* on 5 µl DNA from admission sera using QuantiMix Easy SYG kit (Biotools). The presence of *R. typhi* was examined using the TaqMan real time PCR method of Henry *et al.* [32], targeting *ompB* gene, on 5 µl DNA from admission sera with Platinum Quantitative PCR SuperMix-UDG (Invitrogen).

#### Malaria detection

All patients were tested by HRP-2 *P. falciparum* RDT (Paracheck, Orchid Industries, Goa, India) during the outbreak investigation.

## Results

### Outbreak clinical and epidemiological features

Between 19^th^ November and 18^th^ December 2008, seventy people met our case definition: any person residing in Latsavang Village and experiencing fever and headache with one or more of nausea and vomiting, myalgia and weakness in the previous three weeks, and any children less than on year old presenting with failure to thrive or neurological symptoms in the past three weeks. The median age was 18 years (range, 2 months-64 years); 28 patients (40%) were aged <15 years and 43 (61%) were female. The median (range) duration of illness was 2 (1–7) days. Of the 70 patients, 65 (93%) had fever, 65 (93%) headache, 22 (31%) nausea, 24 (34%) dizziness, 16 (23%) vomiting, 7 (10%) facial erythema, 2 (3%) myalgia, 3 (4%) cyanosis, 3 (4%) convulsions, 2 (3%) conjunctivitis and 1 (1%) had a rash. Of the 42 adult patients, 35 (83%) were farmers.

Six (13.6%) patients died during the outbreak. All were <20 years old and three were infants (1.6–3 months). These infants had no documented fever and presented with central and peripheral cyanosis with convulsions.

The 11 patients who had blood samples taken presented with 1–7 days of fever, headache, nausea and vomiting (Table 1). All of them survived.

### Dengue detection in the outbreak

Amongst the 11 patients with samples, 8 had laboratory evidence of acute dengue infection (Table 2): four tested positive for NS1 antigen and/or by real time PCR and four had serological evidence of recent infection (IgM or high level IgG antibodies). Serotype specific PCR and sequencing confirmed the presence of DENV-1 for all four NS1 and/or PCR positive patients. In addition, for six patients (XB998, XB1000, XB1009, XB1012, XB1013, XB1014) for whom remaining sera were available and placed in cell culture, dengue virus was isolated from the serum of one patient (XB998, NS1+/IgM+/PCR+) and was associated with obvious CPE in Vero cells at day 3 post inoculation and positive PCR detection of DENV-1. No CPE was observed after 7 days for the remaining 5 patients and panflavivirus PCR on cell culture extracts were negative.

**Table 2:** Dengue assay results.

	ELISA											Virus isolation on Vero cells	
	Acute			Convalescent			PCR on acute serum					PCR	
Sample	NS1	IgM	hl.IgG	IgM	hl.IgG	Inter	Den	Den1	PF	nPF	Seqce	Den1	PF
XB998	+	−	−	+	−	Den	+	+	+		Den 1	+	+
XB999	−	+	−	+	−	Den	−	−	−	+	Den 1	N/A	N/A
XB1000	−	+	+	+	+	Den	−	−	−	−		−	−
XB1001	+	+	+	+	+	Den	−	−	−	+	Den 1	N/A	N/A
XB1009	−	−	−	−	−	−	−	−	−	−		−	−
XB1010	−	+	−	N/A	N/A	Den	−	−	−	−		N/A	N/A
XB1011	+	−	−	N/A	N/A	Den	+	+	+		Den 1	N/A	N/A
XB1012	−	−	+	N/A	N/A	Den	−	−	−	−		−	−
XB1013	−	−	−	N/A	N/A	−	−	−	−	−		−	−
XB1014	−	+	−	N/A	N/A	Den	−	−	−	−		−	−
XB1015	−	−	−	N/A	N/A	−	−	−	−	−		N/A	N/A

ELISA results: hl.IgG: High Level IgG; ‘+’: for NS1, IgM or IgG, positive according to the manufacturer's instruction; ‘−’ for negative result; ‘N/A’: no sample available; ‘Inter’: Interpretation, ‘Den’: for evidence of dengue infection. PCR results: ‘Den’: dengue All; ‘Den1’: dengue 1; ‘PF’: panflavivirus; ‘nPF’: heminested panflavivirus; ‘Seqce’: sequencing result of PF or nPF PCR product; ‘+’ in case of dengue genome amplification; ‘−’ in case of no detection of dengue or *Flavivirus* genome.

### Analysis of dengue sequences from the outbreak and elsewhere in Laos

The 18 overlapping DENV-1 PCRs yielded an almost full sequence of the genome of the DENV-1 virus infecting two patients from the outbreak, XB998 (10,629 bases) and XB1011 (9,823 bases), in addition to two patients from Luang Namtha, Northern Lao PDR, (9,823 bases each), two patients from Salavan, Southern Lao PDR, (9,823 and 10,648 bases respectively) and one patient from Vientiane (10,641 bases) (GenBank accession numbers in Table S2). All sequences were obtained from direct processing of patients' sera and shared a high level of identity at the nucleotide level (>97%, Table S3).

**Figure 2:**
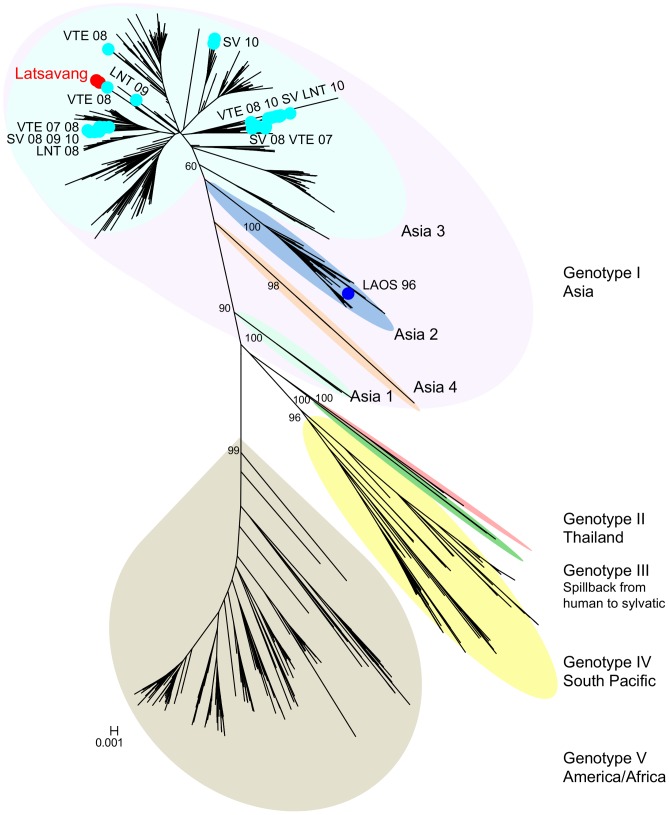
Neighbour-Joining tree of 2,199 dengue 1 envelope gene sequences. Tree produced using Mega 5.05 software with Kimura-2 model. Bootstrap values (in percentage), generated by using 500 replicates, are only indicated for the nodes that define the genotypes, and for clades inside genotype 1. The sequences from the Latsavang outbreak are indicated by red dots. The other Lao strains from this study are indicated by light blue dots while a dark blue dot is used for the 1996 Lao strain. The sequences from Vientiane are indicated by ‘VTE’, the ones from Luang Namtha by ‘LNT’, and the ones from Salavan by ‘SV’; all followed by year of collection.

The tree constructed with 2,199 DENV-1 envelope sequences, including 2 sequences from the Latsavang outbreak, 5 from Luang Namtha, 13 from Salavan and 19 from Vientiane between 2007 and 2010 (GenBank accession number in Table S2), shows (Figure 2) that all Lao strains belong to genotype I.

**Figure 3:**
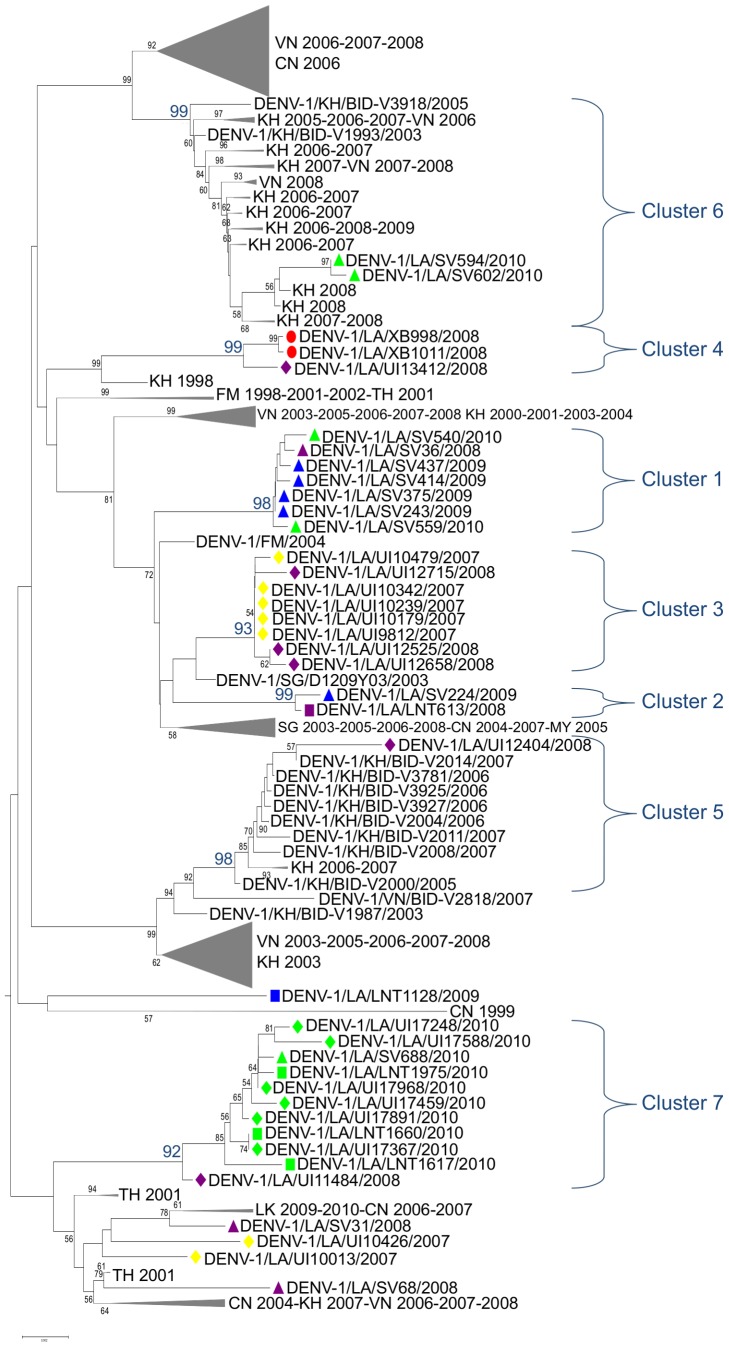
Genotype 1 subtree with genome sequences. Genotype 1 subtree, 843 sequences, from tree performed with the 39 Lao dengue 1 envelope sequences produced in this study aligned with the 1,318 DENV-1 almost complete sequences (>10,000 bases) downloaded from HFVdb. Evolutionary branches that do not include 2007–2010 Lao DENV-1 strains are not shown to increase the legibility. Origin, country (ISO 3166 code) and year of strains within these branches are indicated. The sequences from the Latsavang outbreak (XB) are indicated by red dots. The sequences from Luang Namtha (LNT) are indicated by squares, the one from Salavan (SV) by triangles and the ones from Vientiane (UI) by lozenges. Sequences from 2007 are in yellow, the ones from 2008 (except the ones from Latsavang) are in purple, the ones from 2009 in blue and the ones from 2010 in green. Groups of sequences supported by a high bootstrap value (>90) that contain at least one of Lao sequence are designated as cluster (1 to 7).

Two subtrees, including only genotype I strains, were extracted from the tree constructed with 2,199 DENV-1 envelope sequences and from the tree constructed with the 39 Lao envelope sequences aligned with 1,318 complete sequences. The two subtrees are presented in Supporting Material Figure S1 and Figure 3, respectively. Seven clusters supported by high bootstrap value (>90) can be distinguished in Figure 3. The sequences from the Latsavang outbreak group with a strain from Vientiane of the same period (cluster 4), whereas most of the other Lao strains belong to six other defined clusters. Cluster 1: 7 strains from Salavan 2008, 2009 and 2010. Cluster 2: one strain from Luang Namtha 2008 and one strain from Salavan 2009. Cluster 3: 8 strains from Vientiane 2007 and 2008 and one strain from Vietnam 2007. Cluster 5: one strain from Vientiane 2008 and strains from Cambodia and Vietnam between 2003 and 2007. Cluster 6: 2 strains from Salavan 2010 and strains from Vietnam and Cambodia between 2005 and 2009. Cluster 7: 7 strains from Vientiane 2007 and 2010, 3 strains from Luang Namtha 2010, one strain from Salavan 2010, one strain from China 2010 and one strain from South Korea 2007. Two strains from Vientiane 2007, 2 strains from Salavan 2008 and one strain from Luang Namtha 2009 do not belong to well supported clusters.

Similar topologies were obtained with the Maximum Likelihood method (complete tree and genotype 1 subtree are presented in Supporting Material Figures S2 and S3).

An alignment of 1,325 DENV-1 sequences of 9,756 nucleotides (starting at ORF position 21) was submitted to RDP3 software. The recombination event signals of more than 400 bases with multiple comparison (MC) corrected p-values lower than e-10, obtained by this bioinformatic analysis are displayed in Table S4. Lao dengue strains characterised in this study were not found to be implicated in recombination events. Some putative recombinant strains from China (numbers 1, 4–9, 11,12, and 14 in Table S4) were identified, as described by Wu *et al*. [33]. The other recombination events (numbers 2, 3, 10, and 13) were further investigated by phylogenetic analysis presented in Supporting Material Figure S4.

### Other aetiological investigations for the outbreak

Anti-JEV IgM ELISA, HRP-2 *P. falciparum* RDT and PCR assays for *O. tsutsugamushi*, and *R. typhi* were negative for all samples tested from Latsavang. Sera from 8 patients were tested by IFA for anti-*O. tsutsugamushi* antibodies in acute and, for 6 patients in convalescence samples. All samples had IgM and IgG titres >1∶400 and titres were very high, between 1∶1600 and 1∶3200 (Table S5). No sera were left for IFAs for three patients. There were no significant differences between the acute and convalescent anti-*O. tsutsugamushi* antibody titres. IFA results from 5/6 patients with acute and convalescent sera demonstrated anti-*R. typhi* IgM and IgG titres >1∶400. Two further patients with only acute samples had IgM and IgG titres >1∶400. Titres were generally high, but lower than those against *O. tsutsugamushi*. One patient had acute and convalescent anti-*R. typhi* IgM and IgG titres <1∶400. No sera were left for IFAs for three patients. There were no significant differences between the acute and convalescent anti-*R. typhi* antibody titres.

## Discussion

High IgM and IgG titres against both *O. tsutsugamushi* and *R. typhi* can be detected in healthy rural Lao farmers, presumably reflecting repeated infections in endemic areas (LOMWRU, unpublished data). Therefore, detection of such high titres may be a consequence of repeated or recent rickettsial infections rather than suggesting that *O. tsutsugamushi* and/or *R. typhi* were responsible for the outbreak. This is supported by the lack of change in serological titres (albeit the interval between early and convalescent sera was only 5 days) and the negative PCR for these bacteria (albeit that sera and not buffy coat, were used). Therefore, although rickettsial diseases cannot be excluded as contributing to the outbreak, this aetiology seems unlikely.

In contrast, the evidence that dengue was the predominant pathogen causing the outbreak was supported by the evidence of acute or recent infection for eight of the eleven patients tested. The median (range) patients' age was 15 (5–52) years (n = 11). Of note, all those PCR dengue positive were ≤15 years of age, and all were infected by dengue serotype 1. The clinical presentation was consistent with dengue, but also with many other infections including typhus. The disease was of short duration (1–7 days), consistent with dengue, but no shock or haemorrhage, characteristic of severe dengue, was reported. Nausea and vomiting were frequent. Although not conventionally thought to be major clinical features of dengue, amongst 170 Lao adults with serologically confirmed dengue 44% had nausea and 28% had vomiting [15]. The clinical features of those with and without samples were very similar.

That three infants who died did not have documented fever suggests the possibility they had thiamin deficiency (beriberi), which is not uncommon amongst breastfed Lao infants and is associated with traditional post-partum maternal food avoidance [34]. It is likely that infections may exacerbate thiamin deficiency and it is possible that dengue infection could precipitate fatal thiamin-deficiency heart failure [35]. However no biological confirmation could be provided to support this hypothesis.

Dengue was not suspected to be the responsible pathogen during the outbreak, as in Laos dengue is regarded as an urban disease and incidence is low during the cooler months of November and December. Latsavang is a river bank village of 1,526 inhabitants living in 298 households among maize and paddy fields and surrounded by tropical forest, 120 km as the crow flies from the nearest large town (Xayabury), that has ∼68,000 inhabitants. During the outbreak there were no reports of high dengue incidence in adjacent parts of Laos or Thailand. Daily rainfall data collected in Xayabury town does not show significantly higher rainfall or temperature in November or December 2008 in comparison to the same months in 2009. The mean minimum/maximum monthly temperatures were 17.8/27.9°C and 14.3/26.7°C in November and December, respectively. However, there were many uncovered still water containers and pools in and around the village.

This investigation highlights the difficulty in investigating epidemics of unknown etiology in remote areas. Accessing remote villages, delay in launching investigations, incomplete collection of medical and epidemiological data and of relevant biological samples, shipment conditions, limited size of the population sample studied and absence of negative controls hinder efforts to elucidate aetiology. The results demonstrate the importance of field epidemiology human and financial capacity and standardised sample collection and data recording protocols in developing countries.

This study provided the opportunity to perform the first analyses of the molecular epidemiology of dengue in Laos. Despite endemic dengue infection in Laos, only four partial genomic sequences of dengue virus were available in public databases prior to the current study (including one DENV-1 from 1996). Here, (nearly) complete genomic sequences were established for two patients from the 2008 Latsavang outbreak, but also for two patients from Luang Namtha (2008–2009), two patients from Salavan (2008), and one patient from Vientiane (2008). In addition, complete envelope sequences were produced for a number of Lao DENV-1 positive sera sampled between 2007 and 2010 (Luang Namtha: 3; Salavan: 11; Vientiane: 18).

The tree constructed with 2,199 DENV-1 envelope sequences, shows (Figure 2) the five DENV-1 genotypes previously described [36]. Genotype I includes strains from Southeast Asia, China and East Africa. Genotype II includes Thailand strains from the 1950s and 1960s. Genotype III contains strains from Malaysia, isolated in 1972 from a sentinel monkey and from a dengue patient in 2005 [37]. Genotype III represents spillback from the human into the sylvatic transmission cycle [5]. Genotype IV includes strains from West Pacific Island and Australia. Genotype V includes strains from America, West Africa and limited Asian strains. Within the genotype I, 4 clades have been described [33]: *(i)* a majority of all genotype I strains belongs to the ‘Asia 3’ clade. *(ii)* the ‘Asia 1’ clade includes strains isolated in the 1940's in Japan, Hawai and the USA. *(iii)* the ‘Asia 2’ clade includes mostly Thai (1980–1994), Chinese (2010), Saudi Arabian (2004–2006), Burma (Myanmar) strains (1998–2002), and the only Lao strain (1996) available in GenBank before those sequenced for this study. *(iv)* the ‘Asia 4’ clade includes Chinese strains (1997–1999).

Molecular characterisation of Latsavang DENV-1 outbreak strain shows that it belongs to the ‘Asia 3’ clade of Genotype I, with a genomic sequence very similar to other strains that have caused human infections in the region. Therefore, although the outbreak was in a forested area, it was not caused by a genotypically ‘sylvatic’ dengue virus in the conventional use of the term. Sylvatic dengue strains have been considered to be ecologically and genetically distinct from endemic strains [38], [4], circulating in the forest between non-human primates and arboreal *Aedes* mosquitoes, in contrast to strains responsible for human infections by domestic *Aedes aegypti* and peridomestic *Aedes albopictus* mosquitoes [39], [40]. However, many cases of sylvatic dengue infecting human have been reported [4], [5], [6], [7], [8], [9], [10], [11] and genetically ‘non-sylvatic’, dengue viruses have been detected in wild vertebrates in tropical forests in French Guiana [41]. Little is known about the interplay between anthropogenic and sylvatic transmission cycles and how this may contribute to dengue evolution and maintenance. Using the term ‘sylvatic’, an ecological term, for specific evolutionary groups of strains could therefore be confusing since dengue strains that do not belong to these genotypes may circulate within a sylvatic cycle. Further discussion of the terminology is required with separation of genetic and ecological terms.

Isolating a dengue strain from patients in a rural forest environment during a non-epidemic period raises questions. We identified a closely related strain in Vientiane in late December 2008 (Figure 3) and both strains appear to be descended from viruses described circulating in Thailand, and constitute a lineage that has not been identified anywhere else. The virus may have been brought to Latsavang by someone from Vientiane or elsewhere, provoking an outbreak in a population with limited prior exposure against DENV-1, as suggested by the significant proportion of adults affected (there are no data on incidence of dengue in the Latsavang area before the outbreak). The village is connected to Xayabury via a forest motorable track and the Mekong River is also a transport artery connecting urban centres in north-western Laos. Alternatively, the virus could have been maintained in the Latsavang area through a forest cycle and then transmitted to humans. A major limitation of this investigation was the lack of entomological investigations to determine which mosquito species (*Aedes albopictus*, *Aedes aegypti*, or arboreal *Aedes* species) were dengue vectors in Latsavang. Other limitations include the fact that only 10% of patients were sampled, that there was not a long interval between acute and convalescent samples and that buffy coat blood, for the PCR detection *O. tsutsugamushi* and *R. typhi*, was not available.

Phylogenetic analyses provided the very first clues regarding the molecular epidemiology, dynamics and dispersal of dengue in Laos. First, there is strong evidence that a given strain can be maintained and circulate during consecutive years in specific geographically limited evolutionary clusters. The well-defined phylogenetic cluster 1 (Figure 3), for example, only includes strains from Salavan that were however identified during three consecutive years (2008, 2009, 2010). Similarly, cluster 3 only includes strains from Vientiane in 2007 and 2008. Second, in contrast, it was also observed that a cluster of closely related sequences (cluster 7, Figure 3) was identified in Vientiane, Salavan and Luang Namtha during the same period in 2010. This demonstrates wide geographical viral dispersal, presumably associated with infected patients travelling within Laos. A similar observation is suggested by cluster 4 which includes 2008 Latsavang and Vientiane viruses (see above), and cluster 2 which includes 2008–2009 Salavan and Luang Namtha viruses. This unexplained contrasting epidemiology of clusters deserves attention, suggesting that, despite the rural nature of large tracts of Laos, human activity allows the efficient dispersal of *Aedes*-borne pathogens such as dengue. This illustrated what Stoddard *et al.* recently reported [42], [43] concerning human movement being a key component in determining dengue virus spread. As they suggested, studying individual spatiotemporal movements at fine and broad scales could permit better understanding of the different patterns of dengue transmission reported here and to propose options for dengue control adapted to Laos.

Third, beyond the above-mentioned patterns of local maintenance and dispersal within Lao territory, there is evidence of broad circulation of DENV-1 in the region, crossing national borders. In cluster 5 (Figure 3), a strain from Vientiane 2008 groups with strains from Cambodia and Vietnam isolated between 2003 and 2007. In cluster 6, two strains from Salavan 2010 group with strains from Cambodia and Vietnam isolated between 2005 and 2009. Fourth, recombination analysis, using only bioinformatic tools, did not identify recombinant events amongst DENV-1 Lao strains.

Altogether, these results provide a preliminary, complex picture of dengue epidemiological dynamics in a country in rapid socioeconomic transition. Cornerstones of future investigations may aim for *(i)* a more complete understanding of the entomological and ecological aspects of dengue transmission, dispersal and maintenance; *(ii)* a nationwide collection of population-based sero-epidemiological data, of confirmed infection cases and of viral sequences and (iii) investigation of dengue epidemiology of dengue in rural Laos. It is expected that such information associated with the rapid improvement of diagnostic procedures would allow for a better public health management of dengue in Laos.

## Supporting Information

Figure S1 **Genotype 1 subtree with envelope sequences.** Genotype 1 subtree, 1,627 sequences, from the Neighbour-Joining tree in Figure 2, produced with 2,199 dengue 1 envelope gene sequences. Evolutionary branches that do not include 2007–2010 Lao DENV-1 strains are not shown in order to increase the legibility. Given the high number of sequences displayed in this subtree, origin and date are indicated for group of sequences. Sequences from the Latsavang outbreak are indicated by red dots. Sequences from Luang Namtha (LNT) are indicated by squares, the ones from Salavan (SV) by triangles and those from Vientiane (VTE) by lozenges. Sequences from 2007 are in yellow, the ones from 2008 (except those from Latsavang) are in purple, the ones from 2009 in blue and the ones from 2010 in green. For the other sequences only the country, using ISO3166 code, and the year of origin are indicated. Groups of sequences supported by a high bootstrap value (>90) that contain at least one of Lao sequence are designated as cluster (1 to 7).
(TIF)

Figure S2 **Maximum Likelihood tree of 2,199 dengue 1 envelope gene sequences.** The phylogenetic tree was inferred using the maximum likelihood using RAxML 7.3.0 [44]. The analysis used the GTR model of substitution with a gamma-plus-invariant-sites-distributed rates of change in different sites. Bootstrap values (in percentage), generated by using 500 replicates, are only indicated for the nodes that define the genotypes, and for clades inside genotype 1. The sequences from the Latsavang outbreak are indicated by red dots. The other Lao strains from this study are indicated by light blue dots while a dark blue dot is used for the 1996 Lao strain. The sequences from Vientiane are indicated by ‘VTE’, the ones from Luang Namtha by ‘LNT’, and the ones from Salavan by ‘SV’; all followed by year of collection.
(TIF)

Figure S3 **Genotype 1 subtree with envelope sequences.** Genotype 1 subtree, 1,627 sequences, from the Maximum Likelihood tree in Figure S2, produced with 2,199 dengue 1 envelope gene sequences. Evolutionary branches that do not include 2007–2010 Lao DENV-1 strains are not shown in order to increase the legibility. Given the high number of sequences displayed in this subtree, origin and date are indicated for group of sequences. Sequences from the Latsavang outbreak are indicated by red dots. Sequences from Luang Namtha (LNT) are indicated by squares, the ones from Salavan (SV) by triangles and those from Vientiane (VTE) by lozenges. Sequences from 2007 are in yellow, the ones from 2008 (except those from Latsavang) are in purple, the ones from 2009 in blue and the ones from 2010 in green. For the other sequences only the country, using ISO3166 code, and the year of origin are indicated. Groups of sequences supported by a high bootstrap value (91 for cluster 1, 100 for cluster 2, 3 and 4, 97 for cluster 5, 84 for cluster 6, 97 for cluster 7) that contain at least one of Lao sequence are designated as cluster (1 to 7).
(TIF)

Figure S4 **Neighbour-Joining trees performed for recombination events analyses.** The trees were made using Mega 5.05 with Kimura 2 model from the alignment of the 570 sequences selected by RDP software. Bootstrap values (in percentage) were generated by using 500 replicates. Trees A, C, E, G were done by selecting the sequences inside the breakpoints for the recombinants 2, 3, 10 and 13 respectively. Trees B, D, F, H were done by selecting the sequences outside the breakpoints for the recombinants 2, 3, 10 and 13 respectively. In each tree the recombinant strain is indicated by a blue dot, the major parent by a green dot and the minor parent by a red dot.
(TIF)

Table S1 **Primers used for the amplification of the DENV-1 genome.** Primers DENV1_F2, DENV1_R2, DENV1_F3, DENV1_R3, DENV1_F4, and DENV1_R4, were used for the amplification of the DENV-1 envelope gene.
(DOC)

Table S2 **GenBank accession number of the DENV-1 sequences produced in this study.**
(DOC)

Table S3 **Percentages of identity between DENV-1 genome sequences produced in this study.**
(DOC)

Table S4 **Recombination events detected in the alignment of 1,325 dengue 1 genome sequences using RDP3 software.** Are presented only the recombination events of more than 400 bases with MC corrected p-values lower than e-10. GT: genotype; NS: non-structural; E: envelope; C: capsid; M: membrane.
(DOC)

Table S5 **Assays for bacterial pathogens.** ‘−’ for negative result, ‘N/A’: no sample available.
(DOC)

Text S1 **GenBank accession number of the dengue 1 virus genome sequences used for the design of the primers.**
(DOC)
