# Supplementary figures and images for: An Epidemic of Dengue-1 in a Remote Village in Rural Laos
Source: PLoS Negl Trop Dis. 2013 Aug 8;7(8):e2360. doi: 10.1371/journal.pntd.0002360 (PMC3738459; doi:10.1371/journal.pntd.0002360)

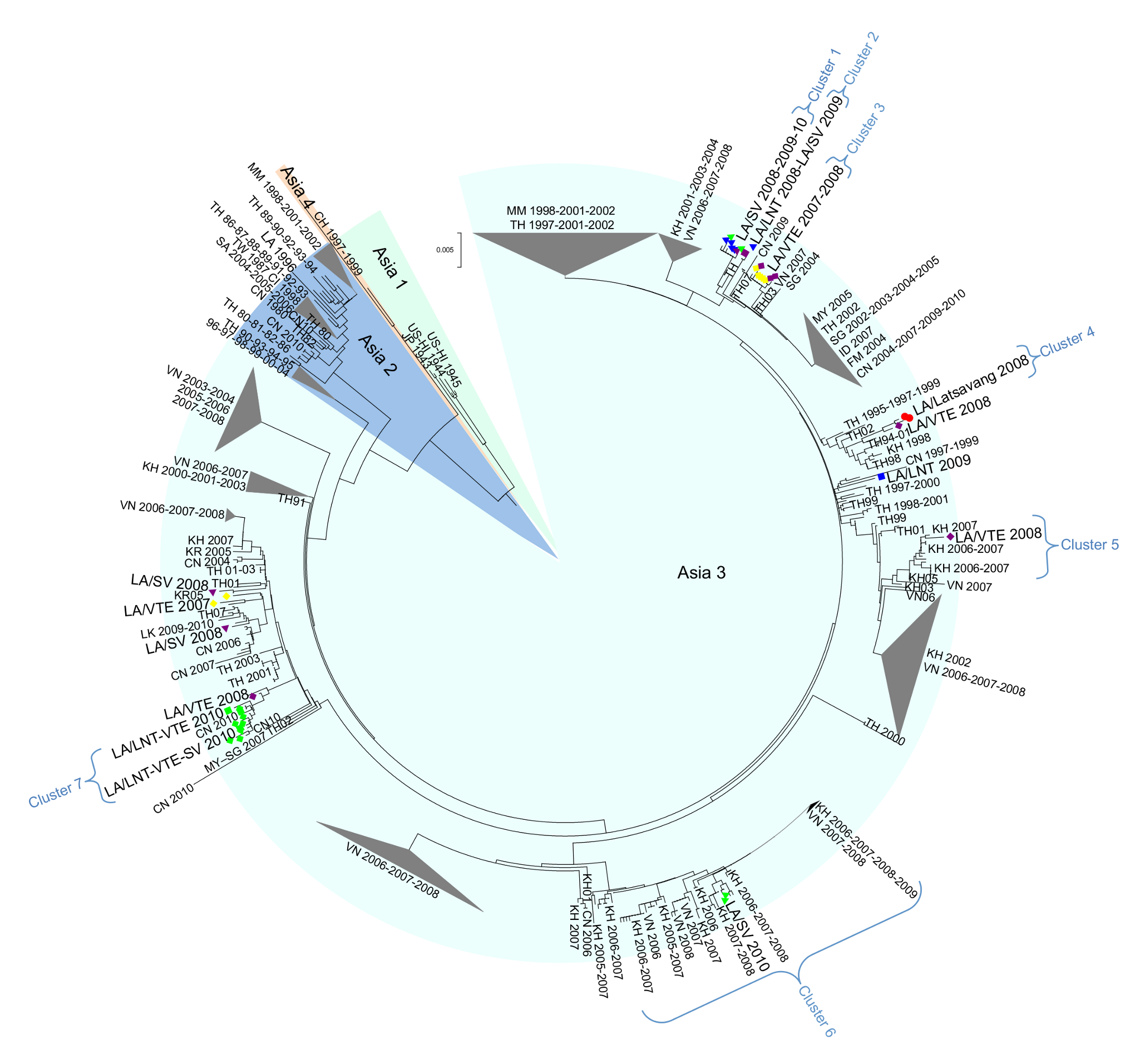

Supplement: Figure S1 — Genotype 1 subtree with envelope sequences. Genotype 1 subtree, 1,627 sequences, from the Neighbour-Joining tree in Figure 2, produced with 2,199 dengue 1 envelope gene sequences. Evolutionary branches that do not include 2007–2010 Lao DENV-1 strains are not shown in order to increase the legibility. Given the high number of sequences displayed in this subtree, origin and date are indicated for group of sequences. Sequences from the Latsavang outbreak are indicated by red dots. Sequences from Luang Namtha (LNT) are indicated by squares, the ones from Salavan (SV) by triangles and those from Vientiane (VTE) by lozenges. Sequences from 2007 are in yellow, the ones from 2008 (except those from Latsavang) are in purple, the ones from 2009 in blue and the ones from 2010 in green. For the other sequences only the country, using ISO3166 code, and the year of origin are indicated. Groups of sequences supported by a high bootstrap value (>90) that contain at least one of Lao sequence are designated as cluster (1 to 7). (TIF) [file pntd.0002360.s001.tif]

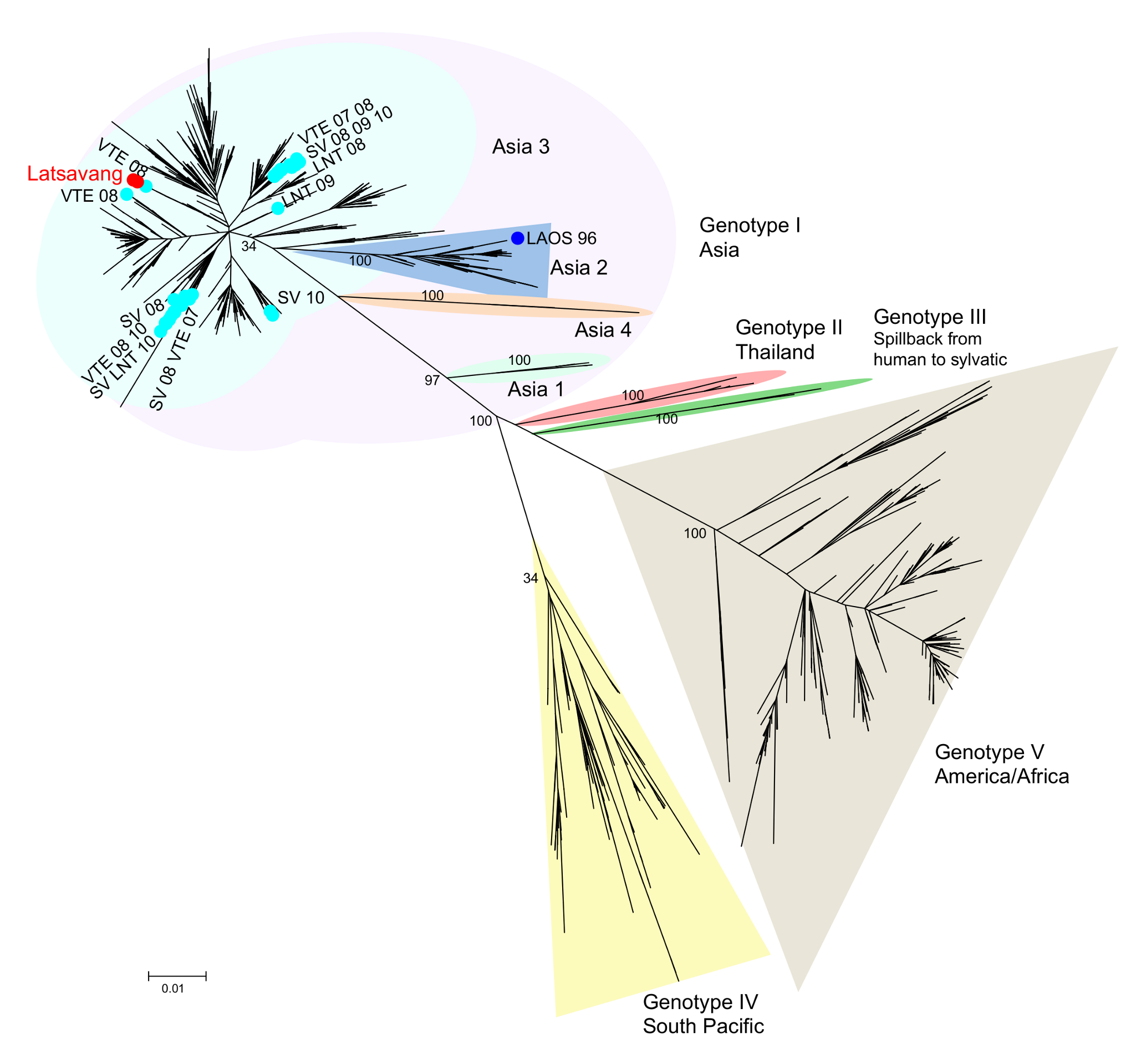

Supplement: Figure S2 — Maximum Likelihood tree of 2,199 dengue 1 envelope gene sequences. The phylogenetic tree was inferred using the maximum likelihood using RAxML 7.3.0 [44]. The analysis used the GTR model of substitution with a gamma-plus-invariant-sites-distributed rates of change in different sites. Bootstrap values (in percentage), generated by using 500 replicates, are only indicated for the nodes that define the genotypes, and for clades inside genotype 1. The sequences from the Latsavang outbreak are indicated by red dots. The other Lao strains from this study are indicated by light blue dots while a dark blue dot is used for the 1996 Lao strain. The sequences from Vientiane are indicated by ‘VTE’, the ones from Luang Namtha by ‘LNT’, and the ones from Salavan by ‘SV’; all followed by year of collection. (TIF) [file pntd.0002360.s002.tif]

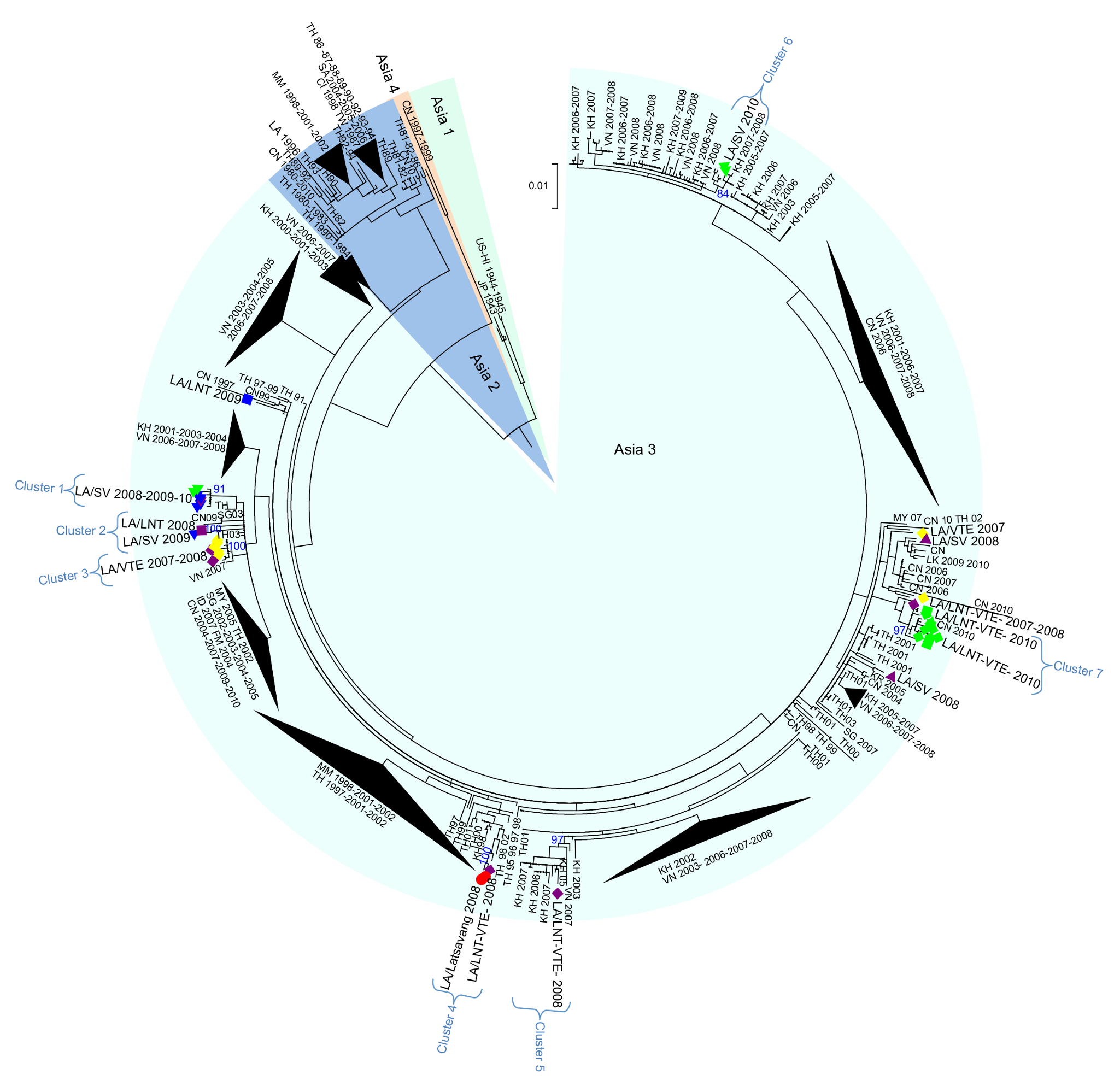

Supplement: Figure S3 — Genotype 1 subtree with envelope sequences. Genotype 1 subtree, 1,627 sequences, from the Maximum Likelihood tree in Figure S2, produced with 2,199 dengue 1 envelope gene sequences. Evolutionary branches that do not include 2007–2010 Lao DENV-1 strains are not shown in order to increase the legibility. Given the high number of sequences displayed in this subtree, origin and date are indicated for group of sequences. Sequences from the Latsavang outbreak are indicated by red dots. Sequences from Luang Namtha (LNT) are indicated by squares, the ones from Salavan (SV) by triangles and those from Vientiane (VTE) by lozenges. Sequences from 2007 are in yellow, the ones from 2008 (except those from Latsavang) are in purple, the ones from 2009 in blue and the ones from 2010 in green. For the other sequences only the country, using ISO3166 code, and the year of origin are indicated. Groups of sequences supported by a high bootstrap value (91 for cluster 1, 100 for cluster 2, 3 and 4, 97 for cluster 5, 84 for cluster 6, 97 for cluster 7) that contain at least one of Lao sequence are designated as cluster (1 to 7). (TIF) [file pntd.0002360.s003.tif]

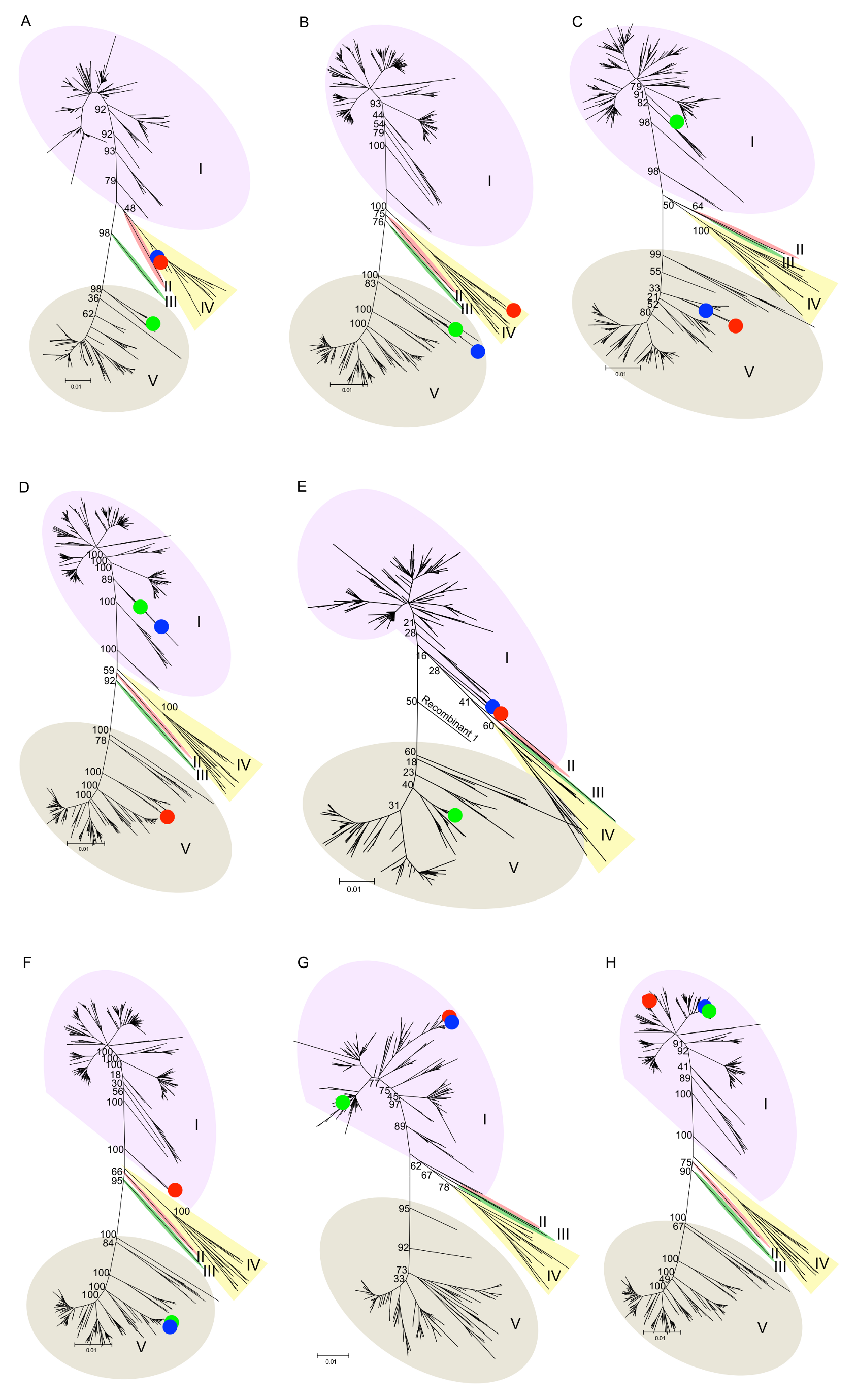

Supplement: Figure S4 — Neighbour-Joining trees performed for recombination events analyses. The trees were made using Mega 5.05 with Kimura 2 model from the alignment of the 570 sequences selected by RDP software. Bootstrap values (in percentage) were generated by using 500 replicates. Trees A, C, E, G were done by selecting the sequences inside the breakpoints for the recombinants 2, 3, 10 and 13 respectively. Trees B, D, F, H were done by selecting the sequences outside the breakpoints for the recombinants 2, 3, 10 and 13 respectively. In each tree the recombinant strain is indicated by a blue dot, the major parent by a green dot and the minor parent by a red dot. (TIF) [file pntd.0002360.s004.tif]
