# Supplementary material for: An Epidemic of Dengue-1 in a Remote Village in Rural Laos
Source: PLoS Negl Trop Dis. 2013 Aug 8;7(8):e2360. doi: 10.1371/journal.pntd.0002360 (PMC3738459; doi:10.1371/journal.pntd.0002360)
Supplement: Table S1 — Primers used for the amplification of the DENV-1 genome. Primers DENV1_F2, DENV1_R2, DENV1_F3, DENV1_R3, DENV1_F4, and DENV1_R4, were used for the amplification of the DENV-1 envelope gene. (DOC) [file pntd.0002360.s005.doc]

Table S1: Primers used for the amplification of the DENV-1 genome.

|  |  | **Position on genome (nucleotide)** | |
| --- | --- | --- | --- |
| **Name** | **Sequences (5’-3’)** | **First base** | **Last base** |
| DENV1_F1 | GTGGACCGACAAGAACAGTTTC | 15 | 36 |
| DENV1_R1 | CTTCVACGAAGTCYCTGYTDCCTAT | 950 | 974 |
| DENV1_F2 | AACAAGARCYGARACRTGGATGTC | 748 | 771 |
| DENV1_R2 | TCCGTAGTCDGTCARYTGTATTTC | 1448 | 1471 |
| DENV1_F3 | GTGYGCHAAGTTYAAGTGTGTGAC | 1279 | 1302 |
| DENV1_R3 | TGGTTTTTCYTTRTCAGTRACTAT | 2003 | 2026 |
| DENV1_F4 | ACATTYAAGACAGCYCATGCAAAG | 1649 | 1672 |
| DENV1_R4 | AATTTRTATTGYTCTGTCCARGTRTG | 2495 | 2520 |
| DENV1_F5 | AGGAWTAGGGRTYCTGCTGAC | 2305 | 2325 |
| DENV1_R5 | TYTTRACTTCTATGAANGAGGCTC | 3060 | 3083 |
| DENV1_F6 | CATYTGGGAAGTTGARGAYTATGG | 2872 | 2895 |
| DENV1_R6 | CAGCYARTGTWCCAGTCATSAGCAT | 3566 | 3590 |
| DENV1_F7 | GGRTGYTGGTAYGGYATGGAAAT | 3401 | 3423 |
| DENV1_R7 | ATTCCTTCRTTGAGRGGCCAACTT | 4129 | 4152 |
| DENV1_F8 | GGACTYGCAATGGGYATYATGAT | 3851 | 3873 |
| DENV1_R8 | CCARCTYGGYTCCARYCTCTTC | 4705 | 4726 |
| DENV1_F9 | ATCWGGAGTSYTRTGGGACACAC | 4519 | 4541 |
| DENV1_R9 | TCRGTRAARTGTGCTTCATCCAT | 5369 | 5391 |
| DENV1_F10 | CTTCCAGCCATAGTYCGWGAGGC | 5132 | 5154 |
| DENV1_R10 | GGYTCCTTCCRATTCTYCCTCT | 5894 | 5915 |
| DENV1_F11 | TGGGACTAYGTYGTCACAACAGA | 5726 | 5748 |
| DENV1_R11 | GAARAACARYGTCACTCCACCAGT | 6569 | 6592 |
| DENV1_F12 | CAGCAGGRAGAAGAAGYGTCKCAG | 6363 | 6386 |
| DENV1_R12 | CCATCCYTTRTCRAGTCCCATCAA | 7052 | 7075 |
| DENV1_F13 | ACAGTRGCAGCYAATGAGATGG | 6815 | 6836 |
| DENV1_R13 | GACACTGCRTGTTTGRTTRTYTCTC | 7716 | 7740 |
| DENV1_F14 | GGAGGGATCTCCAGGAAAATT | 7444 | 7464 |
| DENV1_R14 | CTGTYCCRCATGARACCCARTAC | 8224 | 8246 |
| DENV1_F15 | TGTGAYATYGGWGARTCCTCTCC | 8006 | 8028 |
| DENV1_R15 | CCATTGRTTYTCRTCAACGAACAC | 8804 | 8827 |
| DENV1_F16 | CCATCAGGRTCAGCCTCATCYATG | 8507 | 8530 |
| DENV1_R16 | CCTCTCTGRTCWCGYCTGGATAT | 9347 | 9369 |
| DENV1_F17 | ATACTCAGAGRCATATCAAAGAT | 9122 | 9144 |
| DENV1_R17 | TCTCCTGTGGAARTACATCAG | 9860 | 9880 |
| DENV1_F18 | ATACCRCARTGGGAACCYTCAAA | 9641 | 9663 |
| DENV1_R18 | AGAACCTGTTGATTCAACAGCACC | 10712 | 10735 |

Primers DENV1_F2, DENV1_R2, DENV1_F3, DENV1_R3, DENV1_F4, and DENV1_R4, were used for the amplification of the DENV-1 envelope gene.
