# Supplementary material for: An Epidemic of Dengue-1 in a Remote Village in Rural Laos
Source: PLoS Negl Trop Dis. 2013 Aug 8;7(8):e2360. doi: 10.1371/journal.pntd.0002360 (PMC3738459; doi:10.1371/journal.pntd.0002360)
Supplement: Table S2 — GenBank accession number of the DENV-1 sequences produced in this study. (DOC) [file pntd.0002360.s006.doc]

**Table S2. GenBank accession number of the DENV-1 sequences produced in this study.**

| **Nucleotide sequence** | | | **Sample collection** | | |
| --- | --- | --- | --- | --- | --- |
| **Accession number** | **Type** | **Length** | **Laboratory number** | **Location** | **Date** |
| KC172829 | Full genome | 10629 | XB998 | Latsavang | 15-Dec-08 |
| KC172830 | Full genome | 9823 | XB1011 | Latsavang | 15-Dec-08 |
| KC172831 | Full genome | 9823 | LNT613 | Luang Namtha | 10-Oct-08 |
| KC172832 | Full genome | 9823 | LNT1128 | Luang Namtha | 16-Sep-09 |
| KC172833 | Full genome | 9823 | SV36 | Salavan | 10-Sep-08 |
| KC172834 | Full genome | 10648 | SV68 | Salavan | 06-Oct-08 |
| KC172835 | Full genome | 10641 | UI13412 | Vientiane | 24-Dec-08 |
| KC182082 | Envelope gene | 1723 | LNT1617 | Luang Namtha | 09-Aug-10 |
| KC182083 | Envelope gene | 1723 | LNT1660 | Luang Namtha | 17-Aug-10 |
| KC182084 | Envelope gene | 1720 | LNT1975 | Luang Namtha | 26-Oct-10 |
| KC182085 | Envelope gene | 1471 | SV31 | Salavan | 09-Sep-08 |
| KC182086 | Envelope gene | 1723 | SV224 | Salavan | 06-May-09 |
| KC182087 | Envelope gene | 1723 | SV243 | Salavan | 18-May-09 |
| KC182088 | Envelope gene | 1723 | SV375 | Salavan | 24-Sep-09 |
| KC182089 | Envelope gene | 1723 | SV414 | Salavan | 26-Oct-09 |
| KC182090 | Envelope gene | 1723 | SV437 | Salavan | 12-Nov-09 |
| KC182091 | Envelope gene | 1723 | SV540 | Salavan | 15-May-10 |
| KC182092 | Envelope gene | 1723 | SV559 | Salavan | 14-Jun-10 |
| KC182093 | Envelope gene | 1723 | SV594 | Salavan | 15-Jul-10 |
| KC182094 | Envelope gene | 1717 | SV602 | Salavan | 25-Jul-10 |
| KC182095 | Envelope gene | 1723 | SV688 | Salavan | 27-Oct-10 |
| KC182096 | Envelope gene | 1723 | UI9812 | Vientiane | 14-Jun-07 |
| KC182097 | Envelope gene | 1723 | UI10013 | Vientiane | 13-Jul-07 |
| KC182098 | Envelope gene | 1723 | UI10179 | Vientiane | 01-Aug-07 |
| KC182099 | Envelope gene | 1723 | UI10239 | Vientiane | 10-Aug-07 |
| KC182100 | Envelope gene | 1723 | UI10342 | Vientiane | 23-Aug-07 |
| KC182101 | Envelope gene | 1723 | UI10426 | Vientiane | 01-Sep-07 |
| KC182102 | Envelope gene | 1668 | UI10479 | Vientiane | 07-Sep-07 |
| KC182103 | Envelope gene | 1345 | UI11484 | Vientiane | 06-Feb-08 |
| KC182104 | Envelope gene | 1646 | UI12404 | Vientiane | 08-Jul-08 |
| KC182105 | Envelope gene | 1723 | UI2525 | Vientiane | 22-Jul-08 |
| KC182106 | Envelope gene | 1693 | UI12658 | Vientiane | 11-Aug-08 |
| KC182107 | Envelope gene | 1646 | UI12715 | Vientiane | 18-Aug-08 |
| KC182108 | Envelope gene | 1723 | UI17248 | Vientiane | 05-Jul-10 |
| KC182109 | Envelope gene | 1723 | UI17367 | Vientiane | 13-Jul-10 |
| KC182110 | Envelope gene | 1723 | UI17459 | Vientiane | 20-Jul-10 |
| KC182111 | Envelope gene | 1723 | UI17588 | Vientiane | 28-Jul-10 |
| KC182112 | Envelope gene | 1723 | UI17891 | Vientiane | 18-Aug-10 |
| KC182113 | Envelope gene | 1723 | UI17968 | Vientiane | 23-Aug-10 |
