# Supplementary material for: An Epidemic of Dengue-1 in a Remote Village in Rural Laos
Source: PLoS Negl Trop Dis. 2013 Aug 8;7(8):e2360. doi: 10.1371/journal.pntd.0002360 (PMC3738459; doi:10.1371/journal.pntd.0002360)
Supplement: Table S3 — Percentages of identity between DENV-1 genome sequences produced in this study. (DOC) [file pntd.0002360.s007.doc]

**Table S3**. Percentages of identity between DENV-1 genome sequences produced in this study.

| **Sample collection** | | | **Sequence** | |
| --- | --- | --- | --- | --- |
| **Location** | **Laboratory number** | **Date** | **Length (nucleotides)** | **Percentage of identity with XB998 sequence** |
| Latsavang | XB1011 | 15-Dec-08 | 9823 | 99.96 |
| Luang Namtha | LNT613 | 10-Oct-2008 | 9822 | 97.85 |
| Luang Namtha | LNT1128 | 16-Sep-2009 | 9823 | 97.76 |
| Salavan | SV36 | 10-Sep-2008 | 9823 | 97.92 |
| Salavan | SV68 | 06-Oct-2008 | 10629 | 97.75 |
| Vientiane | UI13412 | 24-Dec-2008 | 10629 | 99.69 |
