# Supplementary material for: An Epidemic of Dengue-1 in a Remote Village in Rural Laos
Source: PLoS Negl Trop Dis. 2013 Aug 8;7(8):e2360. doi: 10.1371/journal.pntd.0002360 (PMC3738459; doi:10.1371/journal.pntd.0002360)
Supplement: Table S4 — Recombination events detected in the alignment of 1,325 dengue 1 genome sequences using RDP3 software. Are presented only the recombination events of more than 400 bases with MC corrected p-values lower than e-10. GT: genotype; NS: non-structural; E: envelope; C: capsid; M: membrane. (DOC) [file pntd.0002360.s008.doc]

**Table S4. Recombination events detected in the alignment of 1,325 dengue 1 genome sequences using RDP3 software.**

|  | | | | |  | | |  | | | **Breakpoint positions on ORF** | | | |
| --- | --- | --- | --- | --- | --- | --- | --- | --- | --- | --- | --- | --- | --- | --- |
| **Recombinant** | | | | | **Major parent** | | | **Minor parent** | | | **Break point in 5’** | | **Break point in 3’** | |
| **no** | **Accession number** | | | **Strain origin** | **Accession number** | **Strain origin** | **GT** | **Accession number** | **Strain origin** | **GT** | **Base** | **Gene** | **Base** | **Gene** |
| 1 | | AY376738 | China 1999 | | AY732482 | Thailand 2001 | I | FJ196846 | China 1995 | IV | 5520 | NS3 | 6548 | NS4A |
| 2 | | EU179860 | Brunei 2005 | | AY732474 | Thailand 1980 | V | EU179861 | Brunei 2006 | IV | 3218 | NS1 | 3640 | NS2A |
| 3 | | A75711 | Singapore 1990 | | AY732480 | Thailand 1994 | I | DQ285559 | Reunion 2004 | V | 976 | E | 2409 | E |
| 4 | | FJ196848 | China 1999 | | AY376737 | China 1997 | I | EF025110 | China 1971 | IV | 913 | E | 1605 | E |
| 5 | | FJ196848 | China 1999 | | FJ196843 | China 2006 | I | FJ196842 | China 2003 | IV | 5769 | NS3 | 6401 | NS4A |
| 6 | | FJ196848 | China 1999 | | EU482488 | Vietnam 2007 | I | FJ196841 | China 2003 | IV | 7815 | NS5 | 8462 | NS5 |
| 7 | | FJ196847 | China 1997 | | AY376737 | China 1997 | I | FJ196846 | China 1995 | IV | 8804 | NS5 | 9756 | NS5 |
| 8 | | AY373427 | China 1995 | | FJ196846 | China 1995 | IV | AY376737 | China 1997 | I | 749 | M | 1167 | E |
| 9 | | FJ196847 | China 1997 | | AY376737 | China 1997 | I | FJ196842 | China 2003 | IV | 5591 | NS3 | 6228 | NS3 |
| 10 | | AY277664 | Argentina 1999 | | AY206457 | Argentine 2000 | V | EU848545 | Hawaii 1944 | I | 850 | E | 1258 | E |
| 11 | | AY376738 | China 1999 | | FJ196848 | China 1999 | I | EF032590 | China 1995 | IV | 21 | C | 466 | C |
| 12 | | EF025110 | China 1971 | | FJ196842 | China 2003 | IV | EF032590 | China 1995 | IV | 5463 | NS3 | 6208 | NS3 |
| 13 | | FJ898399 | Vietnam 2006 | | FJ898402 | Vietnam 2006 | I | GQ199828 | Vietnam 2007 | I | 2258 | NS1 | 2914 | NS1 |
| 14 | | AY376737 | China 1997 | | FJ196847 | China 1997 | I | FJ196848 | China 1999 | I | 1606 | E | 2529 | E |

Are presented only the recombination events of more than 400 bases with MC corrected p-values lower than e-10.

GT: genotype; NS: non-structural; E: envelope; C: capsid; M: membrane
