# Supplementary material for: An Epidemic of Dengue-1 in a Remote Village in Rural Laos
Source: PLoS Negl Trop Dis. 2013 Aug 8;7(8):e2360. doi: 10.1371/journal.pntd.0002360 (PMC3738459; doi:10.1371/journal.pntd.0002360)
Supplement: Table S5 — Assays for bacterial pathogens. ‘−’ for negative result, ‘N/A’: no sample available. (DOC) [file pntd.0002360.s009.doc]

**Table S5. Assays for bacterial pathogens.**

|  | **Scrub typhus (*O. tsutsugamushi*)** | | | | | **Murine Typhus (*R. typhi*)** | | | | |
| --- | --- | --- | --- | --- | --- | --- | --- | --- | --- | --- |
|  | **Acute serum** | | | **Convalescent serum** | | **Acute serum** | | | **Convalescent serum** | |
|  | **PCR** | **IFA titers** | | **IFA titers** | | **PCR** | **IFA titers** | | **IFA titers** | |
| **Sample no** |  | **IgM** | **IgG** | **IgM** | **IgG** |  | **IgM** | **IgG** | **IgM** | **IgG** |
| XB998 | - | 3200 | 3200 | >3200 | >3200 | - | <400 | <400 | <400 | <400 |
| XB999 | - | >3200 | >3200 | >3200 | >3200 | - | 3200 | 1600 | 800 | 400 |
| XB1000 | - | >3200 | 3200 | 3200 | 3200 | - | 800 | 800 | 800 | 800 |
| XB1001 | - | 3200 | 3200 | 3200 | 3200 | - | 800 | 400 | 400 | <400 |
| XB1009 | - | N/A | N/A | N/A | N/A | - | N/A | N/A | N/A | N/A |
| XB1010 | - | 1600 | 3200 | 3200 | 3200 | - | 400 | 800 | 800 | 400 |
| XB1011 | - | N/A | N/A | N/A | N/A | - | N/A | N/A | N/A | N/A |
| XB1012 | - | 3200 | 3200 | 3200 | 3200 | - | 800 | 800 | 800 | 400 |
| XB1013 | - | 1600 | 1600 | N/A | N/A | - | 1600 | 1600 | N/A | N/A |
| XB1014 | - | 3200 | 3200 | N/A | N/A | - | 3200 | 3200 | N/A | N/A |
| XB1015 | - | N/A | N/A | N/A | N/A | - | N/A | N/A | N/A | N/A |

‘-’ for negative result, ‘N/A’: no sample available
