# Supplementary material for: An Epidemic of Dengue-1 in a Remote Village in Rural Laos
Source: PLoS Negl Trop Dis. 2013 Aug 8;7(8):e2360. doi: 10.1371/journal.pntd.0002360 (PMC3738459; doi:10.1371/journal.pntd.0002360)
Supplement: Text S1 — GenBank accession number of the dengue 1 virus genome sequences used for the design of the primers. (DOC) [file pntd.0002360.s010.doc]

**Text S1. GenBank accession number of the dengue 1 virus genome sequences used for the design of the primers.**

AF311958, AF311956, AF513110, AF514883, AF514885, AF514876, AF226685, AF514878, AF514889, AY277666, M87512, AY762084, AF298807, AB074761, AB204803, AB189120, AB195673, AB189121, AB074760, AF309641, AY708047, AY726551, FJ687433, FJ461333, FJ639695, FJ639686, FJ390382, EU687251, FJ410282, FJ461340, FJ176780, DQ193572, EU081276, AB178040, AF298808, AY732483
